# Mutations in *IL36RN* are associated with geographic tongue

**DOI:** 10.1007/s00439-016-1750-y

**Published:** 2016-11-29

**Authors:** Jianying Liang, Peichen Huang, Huaguo Li, Jia Zhang, Cheng Ni, Yirong Wang, Jinwen Shen, Chunxiao Li, Lu Kang, Jie Chen, Hui Zhang, Zhen Wang, Zhen Zhang, Ming Li, Zhirong Yao

**Affiliations:** 1 Department of Dermatology, Xinhua Hospital, Shanghai Jiaotong University School of Medicine, 1665 Kongjiang Road, Shanghai, 200092 China; 2 Department of Stomatology, Xinhua Hospital, Shanghai Jiaotong University School of Medicine, Shanghai, China

## Abstract

Geographic tongue (GT) is a benign inflammatory disorder of unknown etiology. Epidemiology and histopathology in previous studies found that generalized pustular psoriasis (GPP) is a factor associated with GT, but the molecular mechanism remains obscure. To investigate the mechanism of GT, with and without GPP, three cohorts were recruited to conduct genotyping of *IL36RN*, which is the causative gene of GPP. In a family spanning three generations and diagnosed with only GT (“GT alone”), GT was caused by the c.115+6T>C/p.Arg10ArgfsX1 mutation in the *IL36RN* gene. An autosomal dominant inheritance pattern with incomplete penetrance was observed. In the cohort consisting of sporadic cases of “GT alone” (*n* = 48), significant associations between GT and three *IL36RN* variants (c.115+6T>C/p.Arg10ArgfsX1, c.169G>A/p.Val57Ile and c.29G>A/p.Arg10Gln) were shown. In the GPP patient cohort (*n* = 56) and GPP family member cohort (*n* = 67), a significant association between the c.115+6T>C mutation and the simultaneous presence of GPP and GT was observed when compared to the presence of GPP without GT (*P* < 0.05). Biopsies revealed similarities among GT patients with different genotypes (AA, Aa and aa), with the neutrophils prominently infiltrating the epidermis. Western-blot analysis showed that the expression ratio of IL-36Ra/IL-36γ in lesioned tongues with individuals harboring different genotypes (AA, Aa and aa, *n* = 3, respectively) decreased significantly compared to controls (*n* = 3). We describe the mechanism of GT for the first time: some cases of GT are caused by *IL36RN* mutations, while those lacking mutations are associated with an imbalance in expression between IL-36Ra and IL-36γ proteins in tongue tissue.

**Electronic supplementary material:**

The online version of this article (doi:10.1007/s00439-016-1750-y) contains supplementary material, which is available to authorized users.

## Introduction

Geographic tongue (GT [MIM:137400]), also known as benign migratory glossitis (BMG), is a benign inflammatory disorder of the tongue characterized by erythematous lesions of desquamated filiform papillae, which is usually delineated by raised, white, circinate lines (Assimakopoulos et al. 2002). The prevalence of GT varies from 0.2 to 14.29% (Furlanetto et al. 2006), but most surveys show a range between 1.0 and 2.5% (Assimakopoulos et al. 2002) while the etiology of GT remains unknown. Several GT-associated conditions have been reported, such as generalized pustular psoriasis (GPP), heredity, allergies, hormonal disturbances, juvenile diabetes, stress and Down syndrome (Assimakopoulos et al. 2002; Redman et al. 1972). Among them, GPP was commonly proposed to be an associated factor. This was based on the evidence of an increased prevalence of geographic tongue in GPP patients (Morris et al. 1992), similar histological findings in both the skin and tongue lesions (Femiano 2001), and the parallel improvement of both entities after anti-psoriatic treatment (Tholen and Lubach 1987). *IL36RN* was first identified as the causative gene for GPP patients in 2011 and the definition of DITRA (deficiency of interleukin 36-receptor antagonist) was proposed (Marrakchi et al. 2011; Onoufriadis et al. 2011). However, the molecular mechanism of GT has not been described.

In 2013, we reported mutations in *IL36RN* gene in 68 Chinese patients with GPP (Li et al. 2013). At the follow-up visits, a high prevalence of GT in both GPP patients and their non-GPP family members was observed. This interesting phenomenon aroused our interests in studying the relationship between the *IL36RN* gene and expression of GT, in individuals both with, and without, GPP.

## Results

### Pedigree analysis for a Han Chinese family with only GT (“GT alone”)

**Fig. 1:**
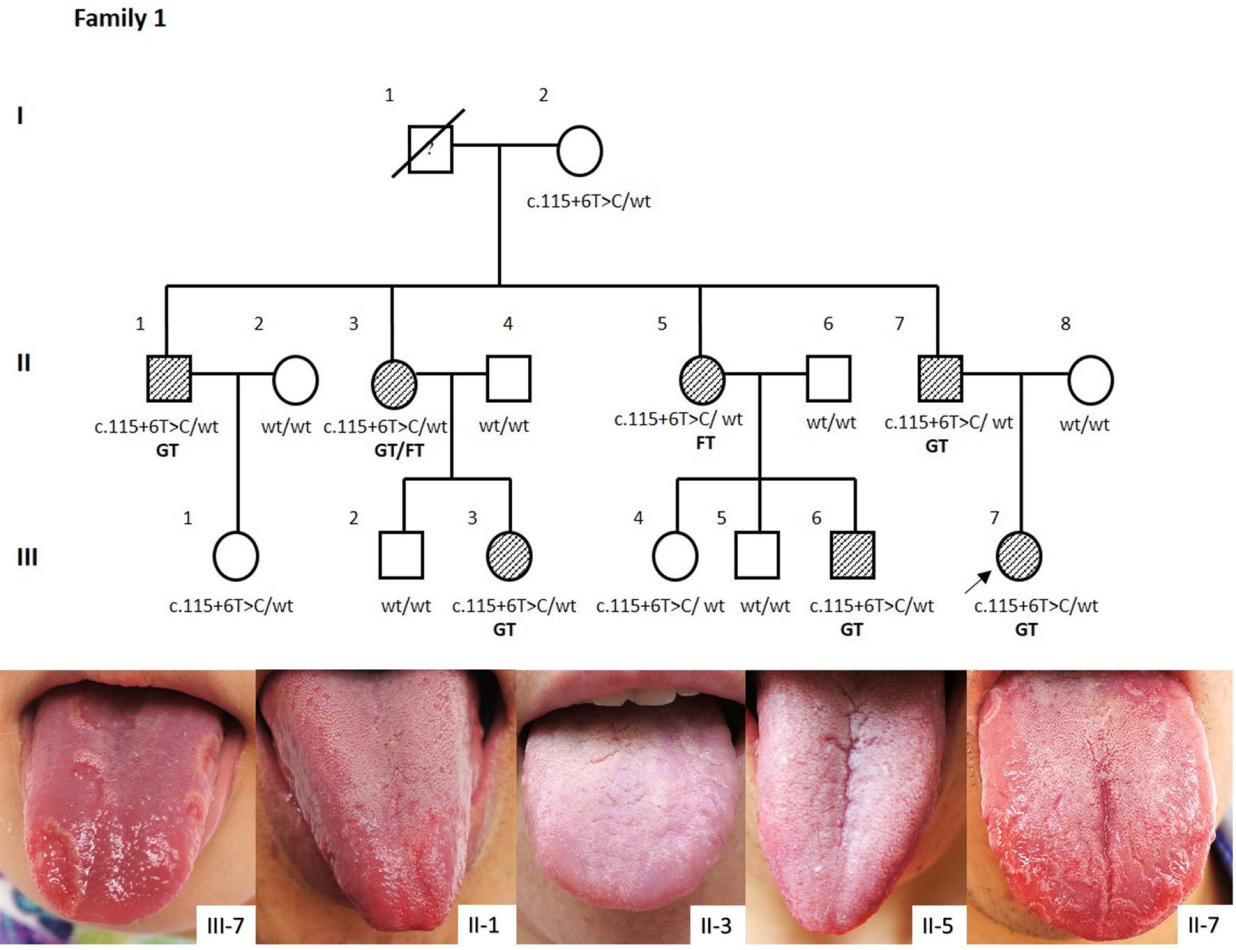
Pedigree of one “GT alone” family. *Filled symbols* denote the severe GT/FT presentations; *cross-hatched symbols* refer to the mild GT/FT presentations; *open symbols* denote absence of GT/FT. Genotypes for c.115+6T>C allele were shown. Wt, wild-type. Family 1: One Chinese Han “GT alone” family without GPP, the proband was a 7-year-old female inpatient with Henoch-Schönlein purpura. GT, which was sustained and aggravated when she got an upper respiratory tract infection, was discovered by her father 3 months after birth. The family extends three generations, with seven members presenting GT or FT. Notably, none of the individuals in this family lacking an *IL36RN* null allele had GT or FT. The clinical manifestations were shown below

In this study, a Han Chinese family manifesting with only GT, or “GT alone”, was recruited (Fig. 1). Here, “GT alone” refers to a GT phenotype lasting more than 6 months without any known DITRA-associated diseases being noted. This family extended three generations and was comprised of 16 individuals, 6 of whom presented with GT and 1 who was shown to have fissure tongue (FT). Sanger sequencing of the *IL36RN* gene revealed that all seven clinically affected members were heterozygous for the c.115+6T>C(p.Arg10ArgfsX1) mutation. Three unaffected individuals also carried this mutation. Notably, none of the individuals lacking *IL36RN* variants had GT or FT. In this family, GT was caused by the *IL36RN* mutation c.115+6T>C(p.Arg10ArgfsX1) present in a heterozygous state. The mutation was inherited in an autosomal dominant manner with an estimated penetrance of 70%.

### Genotyping of sporadic individuals with “GT alone”

A total of 48 sporadic patients with “GT alone” (Supplemental Table 1) and 168 randomly selected controls were recruited to be sequenced for the *IL36RN* gene (Table 1). Three variants, c.115+6T>C(p.Arg10ArgfsX1), c.169G>A(p.Val57Ile) and c.29G>A(p.Arg10Gln) were identified in 16 (33.3%) GT cases, with a combined allele frequency of 0.177. In the control group, the condition of the tongue was firstly determined through physical examination. None of the subjects presented with either the GT or FT lacked any DITRA-associated diseases (0/168). The combined allele frequency of the three variants in the control cohort was 0.015. A highly significant association between the combined *IL36RN* genotypes and GT was observed (odds ratio [OR] 16.30, 95% confidence intervals [CIs] 5.57–47.68, *P* = 2.71E−9). The c.115+6T>C(p.Arg10ArgfsX1) variant was most commonly observed in the “GT alone” cohort, and showed a statistically significant association with GT when compared to the control subjects (OR 10.87, 95% CI 3.60–32.77, *P* = 2.69E−6). The c.169G>A(p.Val57Ile) variant was also significantly associated with GT (*P* = 0.01), whereas c.29G>A(p.Arg10Gln) was not (Table 1).

**Table 1:** Frequency of the *IL36RN* variant alleles in the “GT alone” cohort

Genotype	c.115+6T>C		p.Val57Ile		p.Arg10Gln		Combined genotype	
	Control	GT alone	Control	GT alone	Control	GT alone	Control	GT alone
AA	163	36	168	45	168	47	163	32
Aa	5	11	0	3	0	1	5	15
aa	0	1	0	0	0	0	0	1
Total	168	48	168	48	168	48	168	48
	*P* = 2.69E−6		*P* = 0.01		*P* = 0.222		*P* = 2.17E−9	

GT, geographic tongue; aa, homozygotes; Aa, heterozygotes; AA, wild-type

**Table 2:** The bioinformatics analysis for mutations of *IL36RN*

*IL36RN* nucleotide variations	*IL36RN* amino acid variations	Sequence conservation^a^	Possible impact on structure and function^b^	Protein stability change^c^	Disease association^d^	Effect on protein structure^e^
c.29G>A	p.Arg10Gln	9	Probably damaging (0.997)	Destabilizing (−1.104)	Disease causing	Angles change
c.169G>A	p.Val57Ile	9	Possibly damaging (0.696)	Destabilizing (−0.622)	Polymorphism	Angles change
c.334G>A	p.Glu112Lys	9	Probably damaging (1.000)	Destabilizing (−1.61)	Disease causing	Angles change
c.115+6T>C	p.Arg10ArgfsX1	–	–	–	Disease causing	Truncation

^a^The conservation scores of this site (9-conserved, 1-variable), calculated by ConSurf

^b^Predict possible impact of an amino acid substitution on the structure and function PolyPhen-2, the predict scores are listed in brackets

^c^Protein stability change upon mutation was computed by DUET, scores are listed in brackets, the unit is kcal/mol

^d^The association between the mutations and disease were predicted by MutationTaster

^e^The change on protein structure was predicted by the PyMOL Molecular Graphics system (Version 1.3, Schrodinger LCC)

We used four bioinformatics tools (see “Methods”) to analyze the impact of the three mutations on structure and eventual function of IL-36Ra. The results indicated the pathogenicity of the mutations c.115+6T>C and c.29G>A(p.Arg10Gln). However, the c.169G>A(p.Val57Ile) substitution had a weak effect on the structure and function of IL-36Ra (detailed in Table 2).

**Table 3:** The prevalence of GT in GPPs and family members with c.115+6T>C variant

Genotype	GPP		Family members (GPP with mutations)		Family members (GPP without mutations)	
	With GT	Without GT	With GT	Without GT	With GT	Without GT
AA	13 (59.1%)	9	0 (0%)	3	2 (8.0%)	23
Aa	2 (100%)	0	23 (69.7%)	9	–	–
aa	32 (100%)	0	7 (100%)	0	–	–
Total	47 (83.9%)	9	30 (70.5%)	12	2 (8.0%)	23
	*P* = 2.17E−4		–		–	

GPP, generalized pustular psoriasis; GT, geographic tongue

**Fig. 2:**
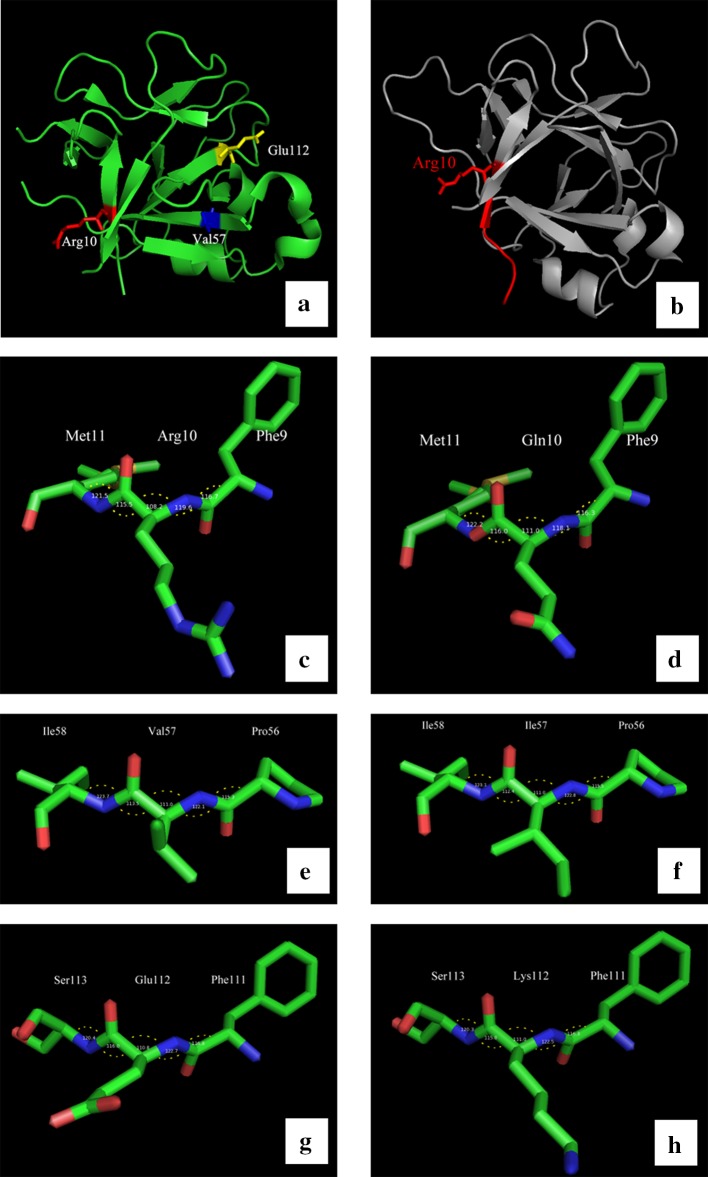
Three-dimensional conformation analysis and structure change of human *IL36RN* protein. **a** The 3D structure of monomer *IL36RN* (PDB id: 4P0L). *Red segment*, *blue segment* and *yellow segment* represented the Arg10 residue, Val57 residue and Glu112 residue, respectively. These sites located on different β-sheets. **b** The mutation, c.115+6T>C, could product a premature translation termination codon (TGA) at the 11th residue. The *gray segment* was the potential truncated part. **c**, **d** The structure and angles of three residues in wild-type *IL36RN* (Phe9-Arg10-Met11) and mutant protein (Phe9-Gln10-Met11). **e**, **f** The structure and angles of three residues in wild-type *IL36RN* (Pro56-Val57-Ile58) and mutant protein (Pro56-Ile57-Ile58). **g**, **h** The structure and angles of three residues in wild-type *IL36RN* (Phe111-Glu112-Ser113) and mutant protein (Phe111-Lys112-Ser113). Angles changed in 3 variants

In three-dimensional conformation analysis, the angles changed in c.29G>A and c.169G>A amino acid substitutions (Fig. 2c–h). Since these angles represented protein backbone, these changes might affect the three-dimensional structure. Moreover, c.115+6T>C mutation has proven to be a truncated protein by Farooq et al. (2013) and Sugiura et al. (2013), three-dimensional conformation analysis for c.115+6T>C was conducted based on the premise of a truncated protein and showed the truncating was caused by a premature translation termination codon (TGA) at the 11th residue (Fig. 2b). Due to the lost of the functional domain, the function of IL-36Ra could be impaired.

Above all, the association between “GT alone” and these *IL36RN* variants suggests that they are genetic risk factors for “GT alone”.

**Fig. 3:**
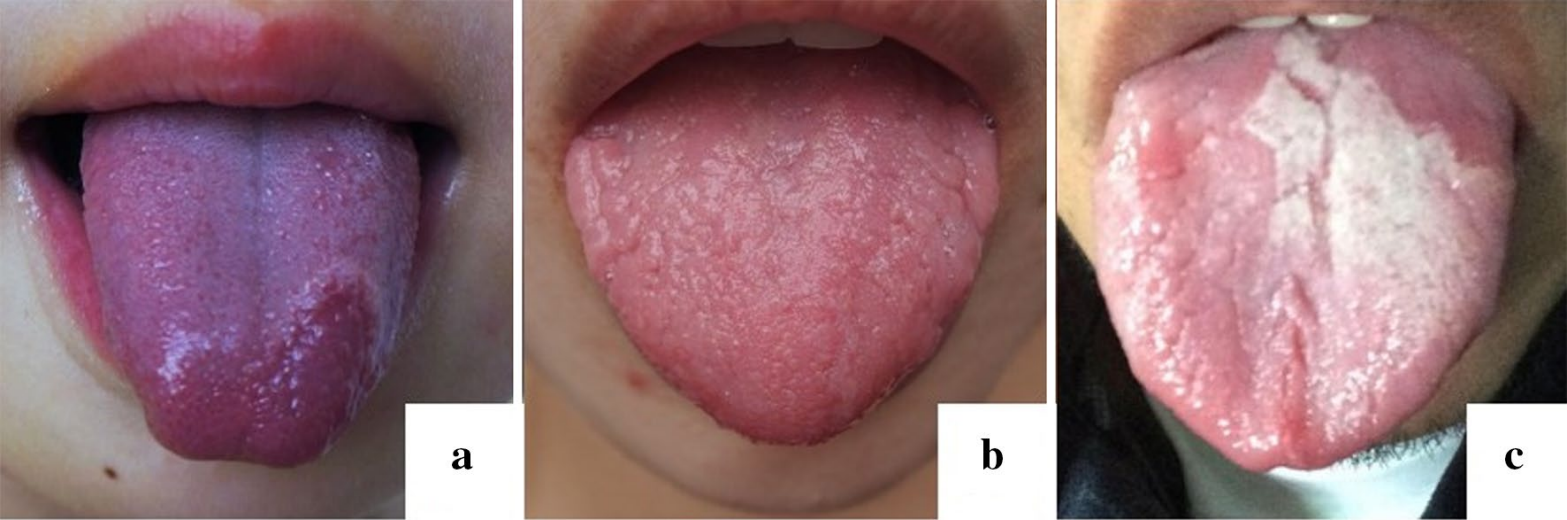
The clinical features in sporadic GT alone patients. **a** GT alone with “Aa” in sporadic cohort. **b** GT alone with “aa” in sporadic cohort. **c** Reexamining the tongue condition of a control with “aa” we recruited in 2013, it was found that he was affected by severe GT

Notably, a 21-year-old c.115+6T>C homozygous female was identified in the “GT alone” cohort. Although manifesting with a severe condition of GT with FT (Fig. 3b), this patient was never affected by GPP or any other DITRA-associated disease.

### Genotype and phenotype analysis of GT for inpatients of GPP and family members of GPP probands

**Figs. 4 and 5:**
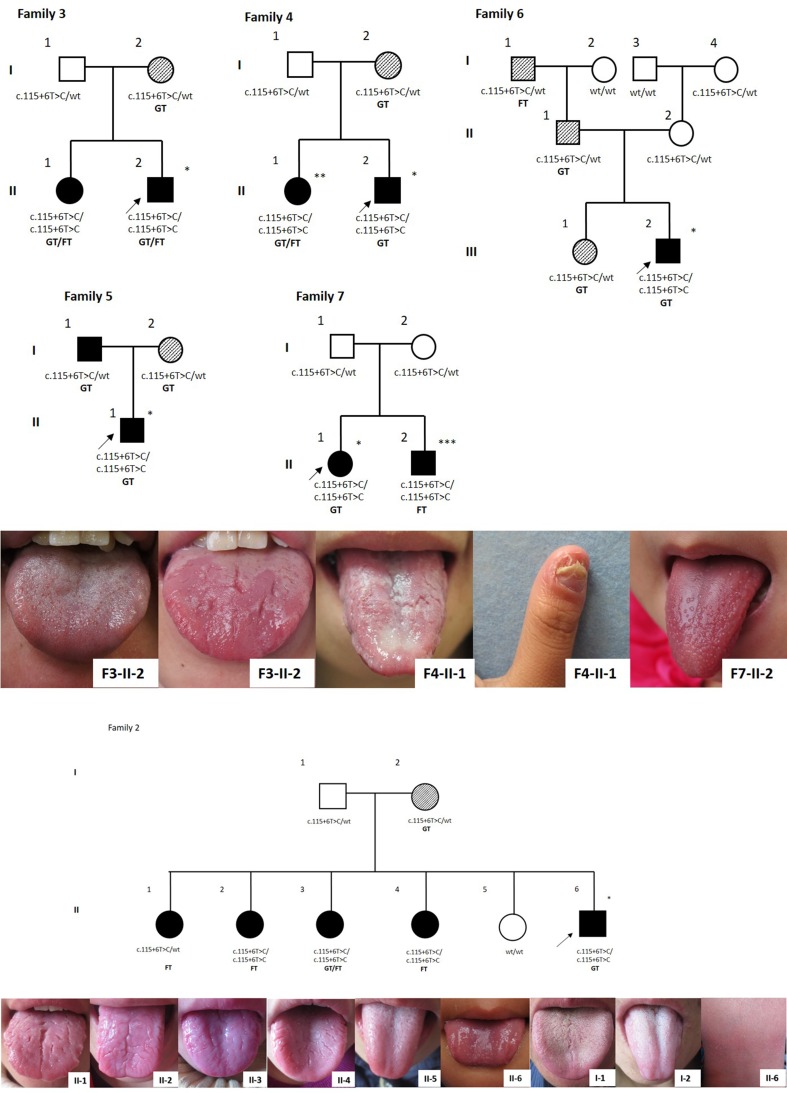
Six typical families affected with GPP and GT. *Single asterisk* denotes individuals with GPP; *double asterisk* denotes ACH; *triple asterisk* denotes fungiform papilla hyperplasia. *Filled symbols* denote the severe GT/FT presentations; *cross-hatched symbols* refer to the mild GT/FT presentations; *open symbols* denote absence of GT/FT; Genotypes for c.115+6T>C alleles are shown. Wt, wild-type. Family 2–7 were typical or remarkable pedigrees from the families with GPP that were recruited and a semi-dominant inheritance pattern was shown. Homozygotes had GPP or severe sustained “GT alone”, most heterozygotes had milder “GT alone” which was inclined to be self-healing, while some of the heterozygotes were unaffected. GT and FT could coexist in the same family. Remarkably, in family 2, the male homozygous proband (family 2; II-6) was affected with severe GPP and GT; however, the female homozygous siblings (family 2; II-2, 3, 4), who ranged from 7 to 23 years of age, were affected by severe “GT alone” and were never affected with GPP. In family 3, the withdrawal of glucocorticoid treatment after 6 months in an “aa” GPP patient revealed the GT phenotype (family 3-II-2). Family 4 showed that ACH (II-1), GPP(II-2), and GT/FT could coexist in the same family. Specifically in family 7, a sustained condition of fungiform papilla hyperplasia(II-2) was seen in a female homozygote without being affected by GPP

The tongue phenotypes and associated genotypes of the *IL36RN* gene was analyzed in 56 in-patients with GPP and 67 family members of the GPP probands (detailed in Supplemental Table 1). In total, 34/56 GPP patients (60.7%) were identified with *IL36RN* mutations. Among them, 31 were homozygous for c.115+6T>C, 1 was compound heterozygous for c.115+6T>C and p.Glu112Lys, and 2 were heterozygous for c.115+6T>C. All 34 GPP patients with *IL36RN* mutations presented with GT. As for the severity of the GT lesions (the definitions of “severe” and “mild” were presented in diagnostic criteria in “Methods”), 31/34 homozygotes and one compound heterozygote (c.115+6T>C/Glu112Lys) were severely affected (Table 3; Fig. 4). The heterozygous GPP patient was accompanied with a mild condition of GT (Supplemental Table 1). On the other hand, 22/56 GPP patients did not harbor any mutations in *IL36RN*. Of these 22 GPP patients, 13 (59.09%) had GT (9 severe, 4 mild), and 9 (40.91%) had a tongue that appeared to be normal (Table 3). A significant association between the c.115+6T>C mutation and the presence of GPP with a GT phenotype was observed when compared to those presenting with GPP but lacking a diagnosis of GT (*P* = 2.17E−4). As for the family members of the GPP probands, 42 family members with *IL36RN* mutations were recruited. Notably, 7/42 were homozygous for c.115+6T>C, all of whom presented with “severe” GT (Fig. 4, F4-II-1, F3-II-1, F7-II-2; Fig. 5, Family2-II-2, II-3, II-4). This included one patient who also presented with acrodermatitis continua of hallopeau (ACH) (Fig. 4, F4-II-1). A total of 32/42 individuals were heterozygous for c.115+6T>C. Among the heterozygous cases, 23/32 (71.9%) had “GT alone” (16 mild, 7 severe), and 9 (28.1%) were unaffected. GT was not found in three wild-type individuals. A total of 13 families encompassing 25 family members were recruited from GPP probands lacking *IL36RN* mutations. Of these 25 individuals, only 2 (8.0%) had “GT alone”.

### Dorsal lingual mucosa biopsy and western-blot analysis for GT patients with different genotypes

**Fig. 6:**
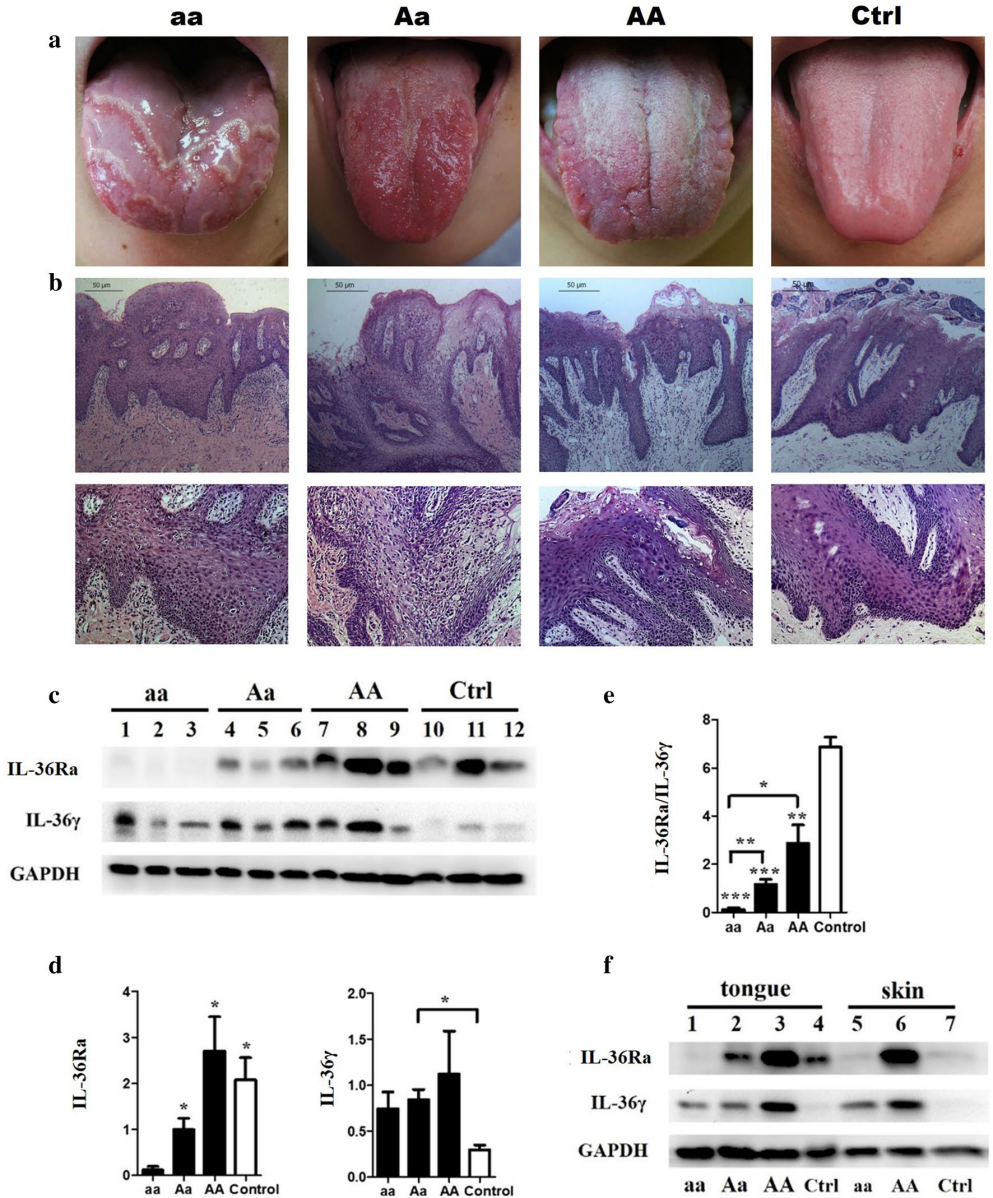
Differences in clinical features, histopathologic characteristics and IL-36Ra/IL-36γ protein expression among GT cases with different genotypes. **a** GT with different genotypes (patient No. 11, No. 82, No. 114 from *left to right*, and a healthy control on *right*, detailed in supplemental Table 1). **b** Biopsy of lesioned tongue tissue shows neutrophil infiltration in epidermis. **c**, **d**, **f** The expression of IL-36Ra and IL-36γ in lingual mucosal tissue of GT patients with different genotypes, aa, homozygotes of c.115+6T>C mutations simultaneously affected with GT and GPP; Aa, c.115+6T>C heterozygotes with GT alone; AA, wild-type “GT alone” patients. The expression of IL-36Ra in lingual mucosal tissue was negative in the aa group, low in the Aa and high in AA groups. No significant differences in expression levels were found among the Aa, AA and control groups. Compared to the “aa” genotypes, those three groups were all significantly increased expressed (specimen 1 from patient No. 11; 2: No. 14; 3: No. 17; 4: No. 82; 5: No. 74; 6: No. 60; 7: No. 173; 8: No. 114; 9: No. 116). **d** IL-36γ is expressed in both healthy controls and all the GT patients with different genotypes. No significant differences were found among GTs of different genotypes. Compared with healthy controls, the protein expression ratio between IL-36Ra and IL-36γ (IL36-Ra/IL-36γ) in lesioned lingual mucosa from GT patients with different genotypes (AA, Aa and aa) were significantly decreased (*n* = 3). Additionally, IL-36Ra/IL-36γ was significantly increased in Aa and AA genotypes in contrast to the aa genotype (*P < 0.05, **P < 0.01, ***P < 0.001). **e** The expression of IL-36Ra and IL-36γ in lingual mucosal tissue of GT and skin tissue of GPP cases from individuals with different genotypes. Skin tissue from a GPP patient with an aa genotype and heathy, wild-type samples served as negative controls for IL-36Ra. Skin tissue from a GPP patient with an AA genotype served as the positive control for IL-36Ra and IL-36γ

To further investigate the histology manifestation and IL-36Ra-associated proteins expression in GT patients with *IL36RN* mutations, histological examination and western-blot analysis was conducted. Dorsal lingual mucosa specimens were obtained from 12 volunteers and included individuals who were: (1) homozygous for c.115+6T>C and manifested with both GT and GPP (*n* = 3), (2) heterozygous for c.115+6T>C and presented with “GT alone” (*n* = 3), (3) found to have a wild-type genotype, but were diagnosed with “GT alone” (*n* = 3), and healthy controls (*n* = 3). The oral histology examinations revealed the similarities in the manifestation of GT relative to the different genotypes (Fig. 6a, b), and showed that the neutrophils prominently infiltrated the epidermis.

Western-blot analysis for the 12 specimens indicated that there is an imbalance in expression between IL-36Ra and IL-36γ in lingual mucosal tissue. This may lead to the GT phenotype despite individuals presenting with different genotypes. Compared to healthy controls, the protein expression ratio between IL-36Ra and IL-36γ (IL-36Ra/IL-36γ) in lesioned lingual mucosa from GT patients with different genotypes (AA, Aa and aa) were significantly decreased (*n* = 3; “aa” vs. “control”, *P* < 0.001; “Aa” vs. “control”, *P* < 0.001; “AA” vs. “control”, *P* < 0.01) (Fig. 6c–e). This suggests that the imbalance between IL-36Ra and IL-36γ expression results in the GT phenotype. Briefly, the over-expression of IL-36γ combined with the insufficient expression of the protective protein IL-36Ra leads to the formation of GT. Furthermore, IL-36Ra/IL-36γ was significantly increased in Aa and AA genotypes in contrast to the aa genotypes (Fig. 6).

However, unlike its negative expression in the skin tissue of healthy controls, IL-36Ra was positively expressed in healthy tongue tissues (Fig. 6f). In lingual mucosal tissue, the IL-36Ra protein was severely impaired in homozygous (aa) subjects, with lower expression levels in heterozygous individuals (Aa) and higher expression in wild-type (AA) subjects manifesting with GT (Fig. 6c). There were no significant differences among “Aa”, “AA” and “control” groups. But when comparing “aa” genotypes to “Aa”, “AA” and “control” groups, these three groups indicated significantly increased IL-36Ra (*P* < 0.05) protein expression (Fig. 6d). IL-36γ is expressed in both healthy controls and all GT subjects, regardless of which genotypes are found. No significant differences were found in IL-36γ expression among individuals with GT and varying genotypes.

## Discussion

Geographic tongue was first reported by Rayer in 1831 (Assimakopoulos et al. 2002), and the etiology of GT still remains unclear. This study reveals the mechanism of GT for the first time. In the “GT alone” multiplex family, GT was caused by autosomal dominant *IL36RN* mutations with incomplete penetrance, but were also identified in GPP patients and their family members. GT is significantly associated with *IL36RN* mutations in the sporadic “GT alone” cohort. For GT with *IL36RN* mutations, we propose that GT can be regarded as a localized manifestation of GPP or a new phenotype of DITRA. A semi-dominant inheritance pattern was observed when GPP was combined with GT as an entity. In contrast to this, and as shown by western-blot analysis, the presence of GT in wild-type individuals were associated with an imbalance in protein expression between IL-36Ra and IL-36γ in tongue tissue.

First of all, GT was confirmed to be caused by an *IL36RN* mutation through studying the “GT alone” family. Genotyping and pedigree analysis of this “GT alone” family, which spans three generations, showed that the causative *IL36RN* mutation (c.115+6T>C/p.Arg10ArgfsX1) existed in a heterozygous state. GT is inherited in an autosomal dominant inheritance pattern with an estimated penetrance of 70% in this family. Next, genotyping of 48 individuals with sporadic cases of “GT alone” showed a highly significant association between the *IL36RN* c.115+6T>C/p.Arg10ArgfsX1 mutant allele and “GT alone” (OR 10.87, 95% CI 3.60–32.77, *P* = 2.69E−6). This further suggested that this *IL36RN* mutation was a genetic risk factor for manifesting “GT alone”. The association between GPP and GT was identified on the basis of an increase in prevalence of GT in both GPP cohort (83.9%, 47/56) and family member cohort (47.8%, 32/67), when compared to the control cohort (0/168) or previous literature reports (ranged from 0.2 to 14.29%) (Furlanetto et al. 2006).

Three mutations, namely, c.115+6T>C/p.Arg10ArgfsX1, c.169G>A/p.Val57Ile and c.29G>A/p.Arg10Gln were identified in “GT alone” patients. The c.115+6T>C mutation was first reported by Farooq et al. (2013) in two Japanese patients with GPP and was demonstrated to lead to the complete lack of exon 3. This results in a premature termination codon, which was supported by reverse transcriptase PCR (RT-PCR) analysis using total RNA from the patients’ skin. Sugiura et al. (2013) also revealed the same result and suggested that p.Arg10ArgfsX1(c.115+6T>C) is one of the founder mutations for GPP in the Japanese population. Furthermore, p.Arg10ArgfsX1(c.115+6T>C) was reported in Chinese (Li et al. 2013, 2014), Korean (Song et al. 2014) and Malay (Setta-Kaffetzi et al. 2013) patients with GPP in homozygous/compound heterozygous, or heterozygous state. In current study, by three-dimensional conformation analysis, the mutation could lead to a truncated protein and lose the functional domain. Predictive results by software also indicate c.115+6T>C is a disease-causing mutation. Then, western-blot analysis showed the negative expression of IL-36Ra both in skin tissue and tongue tissue of GPP patients with homozygous mutation of c.115+6T>C which demonstrated the pathogenicity of the c.115+6T>C mutation in homozygous state. The complete absence of the protein reduced the capacity of inhibiting the IL-36-mediated NF-kB signaling pathway (Tauber et al. 2016). The variants of c.169G>A/p.Val57Ile and c.29G>A/p.Arg10Gln are detected in our GT alone cohort and have not been reported in GPP patients in literatures to our knowledge. Modeling and predictive programs showed the pathogenicity for c.29G>A and the benign characteristic for c.169G>A.

However, about 66.7% (32/48) of the individuals belonging to the sporadic “GT alone” cohort were found to lack *IL36RN* mutations, suggesting that *IL36RN* mutations are not the only disease-causing variants in GT patients. Another disease locus or other factors may be associated with the occurrence of GT.

The inheritance pattern of GPP was autosomal recessive when the *IL36RN* gene was initially identified (Marrakchi et al. 2011). The discovery of GPP patients with single heterozygous mutations (Setta-Kaffetzi et al. 2013; Sugiura et al. 2013; Körber et al. 2013), unaffected individuals with homozygous mutations (Li et al. 2013), as well as the clinical heterogeneity, ranging from mild localized pustular variants to severe systemic GPP (Tauber et al. 2016; Hussain et al. 2015), in homozygous/compound heterozygous states lead to confusion of the inheritance mode of GPP (Capon 2013). Hypotheses of a modifying gene, a second gene locus, tri-allelic disease inheritance patterns, epigenetic events and environmental factors were proposed to explain these confusing phenomena (Farooq et al. 2013; Tauber et al. 2016). Tauber et al. (2016) assessed the functional impact of different *IL36RN* mutations by using site-directed mutagenesis and expression in HEK293T cells, and differentiated null mutations from hypomorphic mutations. Null mutations with complete absence of IL-36Ra were associated with severe clinical phenotypes, while hypomorphic mutations with decreased or unchanged protein expression were identified in both localized and generalized variants, and was thought to account for the clinical heterogeneity. In this study, the correlation between the same mutation (c.115+6T>C/p.Arg10ArgfsX1) with different state (Aa, aa, AA) and clinical phenotypes was analyzed. In the GPP families, homozygotes or compound heterozygotes had GPP or severe “GT alone”, most heterozygotes had milder “GT alone”, and some heterozygotes were unaffected (Figs. 4, 5). The inheritance pattern in our multiplex “GT alone” family was autosomal dominant (Fig. 1). Above all, we propose that GT is a localized manifestation of GPP, and the inheritance pattern of GPP combined with GT is semi-dominant, in that homozygotes or compound heterozygotes had a severe phenotype, whereas heterozygote either had a milder manifestation or no disease phenotype (Palmer et al. 2006). A semi-dominant genetic model explains the phenomenon of pathogenic heterozygotic and non-pathogenic homozygotic in GPP. This reminded us of “the two normal controls of c.115+6T>C variant in a homozygous state” we have previously reported (Li et al. 2013). We reexamined one of the homozygous normal control we managed to contact and found the condition of severe GT (Fig. 3c). The importance of tongue condition examination for GPP family members should be emphasized, while the GT/FT conditions were sometimes ignored in previous literatures about DITRA. Genetic counseling and genotyping should be carried out for GT-affected couples. However, the pathophysiological mechanism of homozygous alleles manifested in localized GT or severe systemic GPP (family 2), and the same heterozygous allele with variable clinical manifestations still remains unclear.

Secondly, we prove that the imbalance of IL-36Ra and IL-36γ is associated with GT of different genotypes (AA, Aa and aa) by western-blot analysis semi-quantitatively. IL-36Ra, encoded by the *IL36RN* gene, can block the downstream inflammatory signal pathway (NF-κB and MAP kinases) which is activated by IL-36 (IL-36α, IL-36β and IL-36γ) through competitively binding to the IL-36 receptor (IL-36R) (Dietrich and Gabay 2014). IL-36Ra, IL-36 and IL-36R are mainly expressed in epithelial tissues including skin, trachea, and esophagus (Marrakchi et al. 2011). Various inflammatory and immunological diseases, such as inflammatory bowel disease (Nishida et al. 2016), systemic lupus erythematosus (Chu et al. 2015), and particularly psoriasis, were discovered to have elevated expression levels of IL-36 cytokines. IL-36γ was regarded as a valuable psoriasis-specific biomarker in both peripheral blood serum and the lesional skin tissue of psoriasis patients (D’Erme et al. 2015). The hypothesis of dysregulation among IL-36 and IL-36Ra cytokines was proposed to explain the predisposition to common forms of psoriasis (Marrakchi et al. 2011). IL-36Ra is a very potent antagonist of the IL-36R-mediated response to IL-36γ at a ratio of IL-36Ra:IL-36γ<1 (Debets et al. 2001). This study proved that the expression ratio of IL-36Ra/IL-36γ in lingual mucosal tissue from GT patients with different genotypes were significantly decreased compared with healthy controls. This indicates that the imbalance between IL-36Ra and IL-36γ expression results in the GT phenotype. Specifically, for the “aa” genotype, the absent expression of IL-36Ra protein resulting from the mutant *IL36RN* causes the sustained phenotype of GT. This is because of the failure of this protein to act as an antagonist for the IL-36 signaling pathway. For the “Aa” genotype, the expression of the IL-36Ra protein is insufficient, leading to the relative over-expression of IL-36γ, and the consequent occurrence of the GT phenotype. As for “AA” patients, although no *IL36RN* variant is found, the expression of IL-36γ is so excessive that the protective protein IL-36Ra is not sufficient to repress the inflammatory activities induced by IL-36γ.

Notably, in normal tongue tissue, the IL-36Ra and IL-36γ proteins are expressed, unlike the absent expression of both proteins in normal skin tissue (Fig. 6f). The difference in expression between skin and tongue tissue may explain the phenomenon that the family members with the “Aa” genotypes in GPP families are inclined to only involve the lingual mucosa rather than skin tissue. Compared to skin tissue, an abundance of the IL-36Ra protective protein is required in tongue tissues in order to antagonize the pre-existing IL-36γ protein expressed. Thus, an insufficient quantity of IL-36Ra is more likely to occur in the tongue of an “Aa” individual, because of the defect in a single *IL36RN* allele.

On the other hand, the manifestation of GT in the absence of mutations, was associated with the inflammatory reaction of increased IL-36γ and a decreased ratio of IL-36Ra/IL-36γ. This may also be induced by infections or immunological diseases. Scarlatina, varicella, alopecia areata, alopecia universalis, atopic dermatitis, localized scleroderma and Henoch-Schönlein purpura are combined with GT in 12 patients belonging to the sporadic “GT alone” cohort (Supplemental Table 1).

In conclusion, our study reveals the mechanism of GT for the first time. Some cases of GT are caused by autosomal dominant *IL36RN* mutations with incomplete penetrance. For GT associated with *IL36RN* mutations, we propose that GT can be classified as a new subtype of DITRA, and that the inheritance pattern of GPP combined with GT is semi-dominant. The higher prevalence of GT in population may facilitate the further investigations of GPP which was constrained by the rarity (Hussain et al. 2015). We emphasize that the condition of tongue should be examined in “Aa” individuals, and GT is an indication for *IL36RN* screening. For GTs without *IL36RN* mutations, we preliminarily confirmed the association with the imbalance expression between IL-36Ra and IL-36γ in tongue tissue.

## Methods

### Subjects

One Han Chinese family with “GT alone” (referring to GT that lasted more than 6 months without any known DITRA-associated diseases), 48 sporadic patients with “GT alone” and 168 randomly selected controls were recruited in the study. An additional 56 inpatients with GPP and 67 family members from GPP probands volunteered to participate in the study. The study was approved by the Medical Ethics Committees of Shanghai Jiaotong University School of Medicine, China. Written informed consent was obtained from all the participants.

### Diagnostic criteria

The diagnosis of GT was simultaneously established by a dermatologist and a dentist. The investigations were performed on the basis of the medical history and clinical examination under natural and artificial light, in accordance with chronic, migratory and macroscopic lesions on the tongue epithelium (Assimakopoulos et al. 2002). The history of “chronic course” was defined as “sustained or intermittent over 6 months” in this study. The lesions were classified into four patterns: (1) patchy areas of desquamated filiform papillae; (2) bordered by an erythematous band of inflammation; and (3) delineated by raised, white, circinate lines. In addition, some patients manifested with fissure tongue (FT). Since the association between GT and FT has been noticed (Assimakopoulos et al. 2002), FT was classified as pattern 4. The pattern number was recorded for all patients (see Supplemental Table 1) and photographs were obtained. Fungal microscopy was performed in each of the GT patients to rule out the presence of candidiasis. The severity was estimated according to the extent of the lesions. More specifically, those lesions exceeding one-third of the surface area of the dorsum were defined as “severe”, while those lesions covering less than one-third of the dorsum were recorded as “mild”.

### *IL36RN* genotyping

Genomic DNA samples were extracted from peripheral blood samples using TIANamp Blood DNA kits (TIANGEN Biotech, Beijing, China) and were amplified by regular polymerase chain reaction (PCR). Primers flanking all exons, as well as intron–exon boundaries of the *IL36RN* gene, were designed. The sequencing of PCR products was conducted on an Applied Biosystems 3730 DNA analyzer (ABI incorporation, Carlsbad, California, USA).

### Dorsal lingual mucosa biopsy

Dorsal lingual mucosa specimens were obtained from 12 volunteers. This included individuals who were homozygous for the c.115+6T>C mutation and presented with both GT and GPP (*n* = 3), individuals who were heterozygous for the c.115+6T>C mutation and presented with “GT alone” (*n* = 3), those with wild-type genotypes manifesting with “GT alone” (*n* = 3), and healthy controls (*n* = 3), respectively. Volunteers are marked with asterisks in Supplemental Table 1.

### Western-blot analysis

Western-blot analysis was performed on the dorsal lingual mucosa specimens from the 12 individuals with IL-36Ra, Rabbit PcAb, IL-36γ, Mouse mAb (Abcam, Cambridge, UK) and anti-GAPDH mouse mAbs. This was followed by HRP-conjugated goat anti-rabbit IgG, goat anti-mouse IgG (H + L) treatment (Beyotime Biological Technology, Jiangsu, China), and ECL detection (Thermo Fisher Scientific, Inc., MA, USA).

### Statistical analysis

Data were analyzed with the SPSS 18.0 software package (SPSS Inc., Chicago, Illinois, USA) and Prism 5 software (GraphPad Software, San Diego, CA).

### Modeling and bioinformatics analysis

To evaluate the impact of mutations on human *IL36RN* protein, the ConSurf program was performed (Ashkenazy et al. 2016) to calculate the conservation scores of these mutation sites. The possible impact of the mutation on the structure and function of *IL36RN* was predicted by an online server, PolyPhen-2 (Adzhubei et al. 2010). The free energy change (ΔΔG) between wild-type and mutation was quantitatively computed by DUET web server (Douglas 2014) to identify protein stability change. MutationTaster (Schwarz et al. 2014) was used to predict the association between mutations and disease. Three-dimensional structure of human *IL36RN* was obtained from RCSB Protein Data Bank (PDB Id: 4P0L), the PyMOL Molecular Graphics system (Version 1.3, Schrodinger LCC) was operated to mutate the amino acid and display the protein structure.

## Electronic supplementary material

Below is the link to the electronic supplementary material.

Supplementary material 1 (DOC 223 kb)

## Abbreviation

GT: Geographic tongue
BMG: Benign migratory glossitis
GPP: Generalized pustular psoriasis
IL-36Ra: Interleukin-36 receptor antagonist
DITRA: Deficiency of interleukin thirty-six–receptor antagonist
ACH: Acrodermatitis continua of hallopeau
FT: Fissure tongue
