# Supplementary material for: Mutations in IL36RN are associated with geographic tongue
Source: Hum Genet. 2016 Nov 29;136(2):241–52. doi: 10.1007/s00439-016-1750-y (PMC5258799; doi:10.1007/s00439-016-1750-y)
Supplement: Supplementary file 1 — Supplementary material 1 (DOC 223 kb) [file 439_2016_1750_MOESM1_ESM.doc]

**Supplemental Table 1 Detailed clinical manifestations and genotypes of GT patients**

| **Genotype** | **No.(patient)**  **-No.(family)** | **Age/Sex** | **GT Pattern** | **Severity** | **DITRA-associated diseases** | **IL36RN mutation** |
| --- | --- | --- | --- | --- | --- | --- |
| **GPP+GT**  **(n=47)** | 1-F2 | 2/M | 123 | severe | GPP | c.115+6T>C/c.115+6T>C |
|  | 2-F3 | 10/M | 1234 | severe | GPP | c.115+6T>C/c.115+6T>C |
|  | 3-F4 | 11/M | 123 | severe | GPP | c.115+6T>C/c.115+6T>C |
|  | 4-F5 | 2/M | 123 | severe | GPP | c.115+6T>C/c.115+6T>C |
|  | 5-F6 | 3/M | 123 | severe | GPP | c.115+6T>C/c.115+6T>C |
|  | 6-F7 | 7/M | 23 | severe | GPP | c.115+6T>C/c.115+6T>C |
|  | 7-F8 | 5/M | 45 | severe | GPP | c.115+6T>C/c.115+6T>C |
|  | 8-F9 | 1/M | 123 | severe | GPP | c.115+6T>C/c.115+6T>C |
|  | 9-F10 | 9/M | 1 | mild | GPP | c.115+6T>C/- |
|  | 10-F11 | 4/M | 2345 | severe | GPP | c.115+6T>C/c.115+6T>C |
|  | 11*-F12 | 11/F | 12345 | severe | GPP | c.115+6T>C/c.115+6T>C |
|  | 12-F13 | 8/M | 1234 | severe | GPP | c.115+6T>C/c.115+6T>C |
|  | 13-F14 | 11/M | 1234 | severe | GPP | c.115+6T>C/c.115+6T>C |
|  | 14*-F15 | 8/F | 123 | severe | GPP | c.115+6T>C/c.115+6T>C |
|  | 15-F16 | 3/F | 123 | severe | GPP | c.115+6T>C/c.115+6T>C |
|  | 16-F17 | 1/M | 123 | severe | GPP | c.115+6T>C/c.115+6T>C |
|  | 17*-F18 | 4/M | 12345 | severe | GPP | c.115+6T>C/E112K |
|  | 18 | 18/M | 1234 | severe | GPP | c.115+6T>C/c.115+6T>C |
|  | 19 | 16/M | 234 | severe | GPP | c.115+6T>C/c.115+6T>C |
|  | 20 | 13/F | 4 | severe | GPP | c.115+6T>C/c.115+6T>C |
|  | 21 | 7/M | 123 | severe | GPP | c.115+6T>C/c.115+6T>C |
|  | 22 | 7/M | 1234 | severe | GPP | c.115+6T>C/c.115+6T>C |
|  | 23 | 6/F | 123 | severe | GPP | c.115+6T>C/c.115+6T>C |
|  | 24 | 6/M | 123 | severe | GPP | c.115+6T>C/c.115+6T>C |
|  | 25 | 11/M | 345 | severe | GPP | c.115+6T>C/c.115+6T>C |
|  | 26 | 10/F | 1234 | severe | GPP | c.115+6T>C/c.115+6T>C |
|  | 27 | 11/M | 4 | severe | GPP | c.115+6T>C/c.115+6T>C |
|  | 28 | 4/M | 1235 | severe | GPP | c.115+6T>C/c.115+6T>C |
|  | 29 | 10/M | 12345 | severe | GPP | c.115+6T>C/c.115+6T>C |
|  | 30 | 13/M | 12345 | severe | GPP | c.115+6T>C/c.115+6T>C |
|  | 31 | 8/F | 1234 | severe | GPP | c.115+6T>C/c.115+6T>C |
|  | 32 | 6/M | 45 | severe | GPP | c.115+6T>C/c.115+6T>C |
|  | 33 | 10/M | 4 | severe | GPP | c.115+6T>C/c.115+6T>C |
|  | 34 | 9/M | 123 | mild | GPP | c.115+6T>C/- |
|  | 35-F19 | 24/M | 234 | severe | GPP | - |
|  | 36-F20 | 8/M | 23 | mild | GPP | - |
|  | 37-F21 | 9/M | 234 | severe | GPP | - |
|  | 38 | 7/M | 1 | severe | GPP | - |
|  | 39-F22 | 5/M | 1 | severe | GPP | - |
|  | 40-F23 | 9/M | 4 | severe | GPP | - |
|  | 41-F24 | 14/M | 2345 | mild | GPP | - |
|  | 42-F25 | 19/M | 1234 | severe | GPP | - |
|  | 43 | 6/F | 1 | severe | GPP | - |
|  | 44 | 3/M | 23 | mild | GPP | - |
|  | 45 | 12/F | 4 | severe | GPP | - |
|  | 46 | 4/F | 123 | severe | GPP | - |
|  | 47 | 5/F | 234 | mild | GPP | - |
| **Family members(with mutations) of GPP probands (n=30)** | 48-F2 | ?/M | 4 | mild | - | c.115+6T>C/- |
|  | 49-F2 | ?/F | 123 | mild | - | c.115+6T>C/- |
|  | 50-F2 | 23/F | 4 | severe | - | c.115+6T>C/- |
|  | 51-F2 | 20/F | 4 | severe | - | c.115+6T>C/c.115+6T>C |
|  | 52-F2 | 9/F | 234 | severe | - | c.115+6T>C/c.115+6T>C |
|  | 53-F2 | 7/F | 4 | severe | - | c.115+6T>C/c.115+6T>C |
|  | 54-F3 | 24/F | 1234 | severe | - | c.115+6T>C/c.115+6T>C |
|  | 55-F3 | 48/F | 1 | mild | - | c.115+6T>C/- |
|  | 56-F4 | 14/F | 34 | severe | ACH | c.115+6T>C/c.115+6T>C |
|  | 57-F4 | ?/F | 12 | mild | - | c.115+6T>C/- |
|  | 58-F5 | ?/F | 1 | mild | - | c.115+6T>C/- |
|  | 59-F5 | ?/M | 1 | severe | - | c.115+6T>C/- |
|  | 60*-F6 | 38/M | 1 | mild | - | c.115+6T>C/- |
|  | 61-F6 | 5/F | 2 | mild | - | c.115+6T>C/- |
|  | 62-F6 | ?/M | 4 | mild | - | c.115+6T>C/- |
|  | 63-F7 | 7/F | 45 | severe | - | c.115+6T>C/c.115+6T>C |
|  | 64-F8 | ?/F | 1 | mild | - | c.115+6T>C/- |
|  | 65-F8 | ?/M | 124 | severe | - | c.115+6T>C/c.115+6T>C |
|  | 66-F9 | ?/M | 1234 | severe | - | c.115+6T>C/- |
|  | 67-F9 | ?/F | 123 | mild | - | c.115+6T>C/- |
|  | 68-F10 | 41/M | 1234 | mild | - | c.115+6T>C/- |
|  | 69-F11 | 43/F | 124 | mild | - | c.115+6T>C/- |
|  | 70-F11 | 15/F | 234 | severe | - | c.115+6T>C/- |
|  | 71-F12 | 45/F | 234 | mild | - | c.115+6T>C/- |
|  | 72-F13 | ?/M | 4 | severe | - | c.115+6T>C/- |
|  | 73-F14 | 40/M | 4 | severe | - | c.115+6T>C/- |
|  | 74*-F15 | 39/M | 14 | mild | - | c.115+6T>C/- |
|  | 75-F16 | 33/M | 14 | mild | - | c.115+6T>C/- |
|  | 76-F16 | 32/F | 1 | mild | - | c.115+6T>C/- |
|  | 77-F17 | 29/M | 4 | severe | - | c.115+6T>C/- |
| **Sporadic**  **“GT alone”(n==48)** | 78 | 21/M | 124 | severe |  | c.115+6T>C/c.115+6T>C |
|  | 79-F1 | 5/F | 123 | severe | Allergic purpura | c.115+6T>C/ - |
|  | 80 | 7/M | 1 | mild |  | c.115+6T>C/ - |
|  | 81 | 5/F | 135 | severe |  | c.115+6T>C/ - |
|  | 82* | 7/F | 15 | severe |  | c.115+6T>C/ - |
|  | 83 | 6/F | 1 | mild |  | c.115+6T>C/ - |
|  | 84 | 7/M | 1235 | severe | Varicella | c.115+6T>C/ - |
|  | 85 | 5/M | 1235 | severe |  | c.115+6T>C/ - |
|  | 86 | 4/M | 15 | mild | SSF | c.115+6T>C/ - |
|  | 87 | 18/F | 12345 | severe |  | c.115+6T>C/ - |
|  | 88 | 25/F | 1 | mild |  | c.115+6T>C/ - |
|  | 89 | 28/M | 1234 | severe |  | c.115+6T>C/ - |
|  | 90 | 8/M | 1 | mild |  | V57I/- |
|  | 91 | 8/M | 1 | mild |  | V57I/- |
|  | 92 | 3/F | 1235 | mild |  | V57I/- |
|  | 93 | 4/M | 1235 | mild |  | R10Q/- |
|  | 94 | 33/M | 1 | mild | AD | - |
|  | 95 | 21/M | 14 | mild | AD | - |
|  | 96 | 6/M | 123 | mild |  | - |
|  | 97 | 2/M | 12 | severe |  | - |
|  | 98 | 8/M | 125 | severe |  | - |
|  | 99 | 5/M | 1235 | severe |  | - |
|  | 100 | 2/M | 123 | severe |  | - |
|  | 101 | 7/M | 123 | severe |  | - |
|  | 102 | 3/F | 23 | mild | Alopecia universalis | - |
|  | 103 | 4/F | 23 | mild | Alopecia areata | - |
|  | 104 | 4/F | 1 | severe |  | - |
|  | 105 | 5/M | 123 | severe | allergic purpura | - |
|  | 106 | 8/F | 125 | mild |  | - |
|  | 107 | 6/M | 12 | mild | Varicella | - |
|  | 108 | 36/M | 23 | mild |  | - |
|  | 109 | 8/F | 245 | severe | AD | - |
|  | 110 | 9/M | 123 | mild |  | - |
|  | 111 | 7/F | 4 | mild |  | - |
|  | 112 | 6/F | 12345 | severe |  | - |
|  | 113 | 13/M | 123 | mild |  | - |
|  | 114* | 9/M | 1234 | severe | scleroderma | - |
|  | 115 | 4/F | 1 | mild |  | - |
|  | 116* | 29/F | 1234 | severe |  | - |
|  | 117 | 62/F | 14 | mild |  | - |
|  | 118 | 32/F | 23 | mild |  | - |
|  | 119 | 55/F | 1234 | severe |  | - |
|  | 120 | 5/M | 1 | mild |  | - |
|  | 121 | 4/F | 23 | mild |  | - |
|  | 122 | 4/F | 123 | severe |  | - |
|  | 123 | 7/F | 12 | severe | AD | - |
|  | 124 | 8/M | 1 | mild |  | - |
|  | 125 | 10/F | 1 | mild |  | - |
| **GT from the multiplex “GT alone” family (n=7)** | 126-F1 | 36/M | 1235 | mild |  | c.115+6T>C/ - |
|  | 127-F1 | 43/M | 12 | mild |  | c.115+6T>C/ - |
|  | 128-F1 | 41/F | 1234 | mild |  | c.115+6T>C/ - |
|  | 129-F1 | 1/M | 12 | mild |  | c.115+6T>C/ - |
|  | 130-F1 | 39/F | 4 | mild |  | c.115+6T>C/ - |
|  | 131-F1 | 1/M | 12 | mild |  | c.115+6T>C/ - |
| **GPP without GT(n=9)** | 132-F26 | 6/M | - | - | GPP | - |
|  | 133-F27 | 7/M | - | - | GPP | - |
|  | 134-F28 | 5/M | - | - | GPP | - |
|  | 135 | 7/M | - | - | GPP | - |
|  | 136-F29 | 8/F | - | - | GPP | - |
|  | 137 | 14/M | - | - | GPP | - |
|  | 138-F30 | 8/M | - | - | GPP | - |
|  | 139-F31 | 7/M | - | - | GPP | - |
|  | 140 | 6/M | - | - | GPP | - |
| **nGT from GPP families**  **(n=37)** | 141-F2 | 5/F | - | - | - | - |
|  | 142-F3 | 47/M | - | - | - | c.115+6T>C/- |
|  | 143-F6 | 36/M | - | - | - | c.115+6T>C/- |
|  | 144-F6 | 58/F | - | - | - | - |
|  | 145-F6 | 65/M | - | - | - | c.115+6T>C/- |
|  | 146-F6 | 62/F | - | - | - | - |
|  | 147-F10 | 40/F | - | - | - | c.115+6T>C/- |
|  | 148-F7 | 32/M | - | - | - | c.115+6T>C/- |
|  | 149-F7 | 29/F | - | - | - | c.115+6T>C/- |
|  | 150-F11 | 46/M | - | - | - | c.115+6T>C/- |
|  | 151-F17 | 26/F | - | - | - | c.115+6T>C/- |
|  | 152-F18 | 32/F | - | - | - | c.115+6T>C/- |
|  | 153-F19 | 55/F | - | - |  |  |
|  | 154-F19 | 58/M | - | - |  |  |
|  | 155-F20 | 31/M | - | - |  |  |
|  | 156-F20 | 32/F | - | - |  |  |
|  | 157-F21 | 35/M | - | - |  |  |
|  | 158-F21 | 34/F | - | - |  |  |
|  | 159-F22 | ?/M | - | - |  |  |
|  | 160-F22 | ?/F | - | - |  |  |
|  | 161-F23 | 37/M | - | - |  |  |
|  | 162-F23 | 34/F | - | - |  |  |
|  | 163-F24 | 42/M | 1 | mild |  |  |
|  | 164-F24 | 38/F | - | - |  |  |
|  | 165-F25 | ?/M | - | - |  |  |
|  | 166-F25 | 44/F | - | - |  |  |
|  | 167-F26 | 32/M | - | - |  |  |
|  | 168-F26 | 34/F | - | - |  |  |
|  | 169-F27 | 36/M | - | - |  |  |
|  | 170-F27 | ?/F | - | - |  |  |
|  | 171-F28 | ?/M | - | - |  |  |
|  | 172-F28 | ?/F | - | - |  |  |
|  | 173*-F29 | 38/M | 12 | mild |  |  |
|  | 174-F29 | 38/F | - | - |  |  |
|  | 175-F30 | 37/M | - | - |  |  |
|  | 176-F31 | 35/M | - | - |  |  |
|  | 177-F31 | 36/F | - | - |  |  |
| **nGT from the “GT alone ” family(n=8)** | 178-F1 | 33/F | - | - | - | - |
|  | 179-F1 | 13/F | - | - | - | c.115+6T>C/ - |
|  | 180-F1 | 7/M | - | - | - | - |
|  | 181-F1 | 9/F | - | - | - | c.115+6T>C/ - |
|  | 182-F1 | 1/M | - | - | - | - |
|  | 183-F1 | 18/M | - | - | - | - |
|  | 184-F1 | 42/M | - | - | - | - |
|  | 185-F1 | 64/F | - | - | - | c.115+6T>C/ - |

F1 denoted family 1, and so on;

Pattern of GT: 1, patchy areas of desquamated filiform papillae;

2, bordered by an erythematous band of inflammation;

3, delineated by raised, white, circinate lines;

4 fissure tongue (FT);

5, tongue condition of fungiform papilla hyperplasia.

Severity was estimated: severe, exceeding one-third of the dorsumas;

mild , less than one-third of the dorsum;

SSF, streptococcal scarlet fever; AD, atopic dermatitis.

* marks out the volunteers of lingual mucosa biopsy.
